# Kirenol Inhibits B[a]P-Induced Oxidative Stress and Apoptosis in Endothelial Cells via Modulation of the Nrf2 Signaling Pathway

**DOI:** 10.1155/2021/5585303

**Published:** 2021-04-23

**Authors:** Peramaiyan Rajendran, Abdullah M. Alzahrani, Emad A. Ahmed, Vishnu Priya Veeraraghavan

**Affiliations:** ^1^College of Science, Department of Biological Sciences, King Faisal University, Al Ahsa, 31982., Saudi Arabia; ^2^Department of Molecular Physiology, Zoology Department, Faculty of Science, Assiut University, Egypt; ^3^Department of Biochemistry, Saveetha Dental College, Saveetha Institute of Medical and Technical Sciences, Saveetha University, Chennai 600 077, India

## Abstract

Atherosclerosis is a persistent inflammatory disorder specified by the dysfunction of the arteries, the world's leading cause of cardiovascular diseases. We sought to determine the effectiveness of KRL in B[a]P-induced oxidative stress and programmed cell death in endothelial cells. Western blotting, real-time PCR, DCFH2-DA, and TUNEL staining were performed to detect pPI3K, pAKT, Nrf2, HO-1, NQO-1, Bcl2, Bax, and caspase-3 on the HUVECs. Through the pretreatment of KRL, a drastic enhancement was observed in the cell viability of HUVECs, whereas DNA damage and generation of reactive oxygen species induced by B[a]P was suppressed. KRL's potential use as an antioxidant was observed to have a direct correlation with an antioxidant gene's augmented expression and the nuclear translocation activation of Nrf2, even during the event when B[a]P was found to be absent. In addition, this study proved that the signaling cascades of PI3K/AKT mediated Nrf2 translocation. Activation of suppressed nuclear Nrf2 and reduced antioxidant genes across cells interacting with an LY294002 confirmed this phenomenon. In addition, knockdown of Nrf2 by Nrf2-siRNA transfection abolished the protective effects of KRL on HUVECs cells against oxidative damage. Finally, the expression of apoptotic proteins also supported the hypothesis that KRL may inhibit endothelial dysfunction. This study showed that KRL potentially prevents B[a]P-induced redox imbalance in the vascular endothelium by inducing the Nrf2 signaling via the PI3K/AKT pathway.

## 1. Introduction

As pervasive environmental carcinogens, heterocyclic aromatic hydrocarbons cause genetic damage and endowed with a strongly bioaccumulation attribute. As a PAH (polyaromatic hydrocarbon), benzo[a]pyrene (B[a]P) finds itself in first class of carcinogens issued by the International Agency for Research on Cancer. Exposure to toxic B[a]P mainly takes place because of the existing food chain and cigarettes. As per previous studies, 9.20 ng/day is the maximum exposure of people to this PAH [1]. Meanwhile, its sustained exposure can lead to metastasis as well as angiogenesis in many organs of the human body, such as the stomach, liver, skin, lungs, and colorectal organs [2, 3]. According to a vast body of scholarly research, one of the strongest causes of vascular inflammation is oxidative stress [4]. Previous literature in animal models clearly illustrated that direct exposure to B[a]P can cause including cancers and pulmonary, neurodegenerative, and cardiovascular diseases [2, 5–7]. Atherosclerosis is thought, at least in part, to be an inflammatory mechanism caused by free radicals [8, 9]. In addition to this, B[a]P was shown to stimulate formation of ROS and promote the progression of atherosclerosis [10, 11]. Mechanisms that are redox-sensitive are used to monitor various inflammatory genes and related transcription factors. B[a]P is known to cause atherosclerotic lesions when it comes to animal models [12, 13].

Nrf2 can easily get liberated from “Kelch-like ECH-associated protein 1” (KEAP1) and transport from the cytosol to the nucleus when confronted with oxidative stress, activating antioxidative gene expression including heme oxygenase-1 (HO-1). The phospholipid, namely, phosphatidylinositol 3-kinase (PI3K) super family, belongs to the DNA-dependent protein kinase catalytic subunit (DNA-PKcs). From the previous literature, we can understand that DNA-PKcs and the PI3K/protein kinase B (AKT) signaling pathway have been involved in HO-1 induction with response to PAH-induced oxidative stress [14–16]. AKT's reversible oxidation is its planned mechanism. For this reason, regulating signaling of Nrf2 mediated by PI3/AKT, to control DNA damage induced by ROS, could possibly be helpful in developing new drugs against vascular damages induced by B[a]P. In order to reduce toxicity induced by B[a]P as well as to treat atherosclerosis, natural products serve as important resources to atherosclerosis therapy [17–19]. In this context, kirenol (KRL) is sourced from *Herba Siegesbeckia*, which has historically utilized in China for the treatment of rheumatoid arthritis. KRL exhibits anti-inflammatory and antirheumatic properties [20]. In addition to demonstrating that this compound can lower the pathology of inflammation in model rats, other literature suggests that it can also inhibit TNF-*α* and IL-1*β* production in adjuvant arthritis model animal serum [21–23]. Wu et al. also demonstrated that KRL offers potent cardioprotective effects [24]. Here, we hypothesized that KRL can activate Nrf2 possibly because of the phenolic compounds' antioxidant action against vascular toxicity induced by B[a]P.

## 2. Materials and Methods

### 2.1. Reagents

All reagents (standard) were derived from Sigma-Aldrich unless mentioned otherwise. This study used the following antibodies: antibodies against pAKT, AKT, pPI3K, PI3K, BCL2, BAX, HO-1, NQO-1, pNrf2, actin, and lamin B1 derived from Invitrogen, Waltham, MA-based Thermo Fisher Scientific, Inc., LY294002 (L9908). Meanwhile, caspase-3 (anti-cleaved) (ab32042) antibody was purchased from Abcam (Branford, CT, USA). Lipofectamine 2000 (11668027) and Nrf2 siRNA were procured from Thermo Fisher Scientific, Inc.

### 2.2. Cell Culture

Derived from Shanghai Institute of Cell Biology, the culturing of HUVECs (passage 15) in DMEM was done with heat-inactivated FBS, 100 U/ml penicillin, and 100 U/ml streptomycin. The maintenance of cells was done in 100 mm dishes in CO_2_ humidified atmosphere with 37°C incubation.

### 2.3. Cell Culture Treatment

To examine the protective effect of KRL against oxidative stress, HUVECs cells were treated with 25 *μ*mol of KRL for 2 h before B[a]P (5 *μ*mol); cell lysate was collected for analysis after 24 h.

### 2.4. Intracellular ROS Assay

The HUVECs cells' seeding was undertaken in the eight-well chamber (LabTech). This chamber comprised 10% DMEM by FBS, with a confluence growth of 80%. After the treatment, the mechanism of dichlorofluorescin diacetate (DCFH2-DA) was utilized for washing cells with PBS. In addition, intracellular ROS was measured by a Leica D6000 fluorescence microscope.

### 2.5. Measurement of Nitric Oxide Production

Cells were grown on 35 mm diameter cell culture dishes and then incubated for 6 h with the test compounds or DMSO as a solvent control (0.1%) in Dulbecco's modified Eagle's medium (Invitrogen, Carlsbad, CA, USA). Nitric oxide production was estimated by determination of nitrites/nitrates in the cell culture medium using the Griess reaction. Detection involved the enzymatic conversion of nitrates to nitrites by nitrate reductase (Sigma-Aldrich), followed by colorimetric detection of nitrites as a colored azo dye product. The absorbance was measured at 546 nm using the SPECTRA Rainbow (Tecan, Austria) microplate reader.

### 2.6. Preparation of Cytosolic and Nuclear Extracts

The resuspending of cell pellets was done in buffer I for preparing cytosolic extracts in a period of five minutes. Buffer I consisted of 5 mM KCl, 0.5 mM MgCl_2_, 25 mM HEPES pH 7.9, and 1 mM dithiothreitol. The suspension was then bolstered with buffer II with the same specifications. Thereafter, the suspension was added by NP40, which, in turn, was supplemented by phosphatase/protease inhibitors. The incubation of resultant samples was done for 15 min. These lysates were centrifuged at 2500 rpm for 5 min. For getting rid of residual nuclei, these lysates were centrifuged at the same temperature after which the transferring of supernatants was done to Eppendorf tubes. The rotation of lysates was done at a temperature of 4°C for a period of 1 hour before it was centrifuged at the same temperature for 10 min [25].

### 2.7. Nrf2-siRNA Transient Transfection

During the transfection, the plating of cells was done in six-well plates for reaching a confluency of 40–60%. After that, 500 *μ*l of culture medium (Opti-MEM) was utilized, whereas the cells were transfected by the RNAiMAX transfection reagent. A combination of siRNA100 pM, an Opti-MEM (500 *μ*l), and RNAiMAX reagent was used in a different tube. After transfecting siRNA using Lipofectamine RNAiMax, the instructions of KRL manufacturers were complied with. The incubation of siRNA/RNAiMAX blend was done for a period of 25 min. Subsequently, this solution was added with the cells in the aforementioned plates, following which the solution was finally incubated for a period of 6 hours. This was followed by a culturing of cells at normal temperature after substituting the medium of transfection using a standardised growth medium.

### 2.8. Western Blotting

Following the treatment, the scraping of cells was done before being rinsed inside cold PBS. After that, the preparation of nuclear, total, and cytoplasmic extract was undertaken. In all the samples, Bio-Rad protein assay helped ascertain the concentration of protein and bovine serum albumin (BSA) in order to be used as the standard of reference. On the other hand, equal quantities (50 *μ*g) of protein were resolved using SDS-PAGE (8-15%) before being moved across to nitrocellulose membranes. On the other hand, the membranes were blocked for half an hour at room temperature using 5% skimmed milk, prior to being incubated for a couple of hours using primary antibodies. Subsequently, anti-rabbit secondary antibody or goat anti-mouse (horseradish peroxidase-conjugated) incubated the membranes for the same duration. The membranes were developed with an improved chemiluminescence substrate. Meanwhile, the samples were examined using an LI-COR chemiluminescence imaging system. Finally, the graphs pertaining to the intensities of the densitometric band were made using Image Studio™ Lite software. Normalisation of the untreated control band's intensity was fixed at 1.

### 2.9. TUNEL Assay

The cells were incubated with KRL and/or B[a]P for 24 h at given doses, and TUNEL assay was studied as described previously [26].

### 2.10. Real-Time Polymerase Chain Reaction (RT-PCR)

The treatment of HUVECs cells was done using B[a]P and/or KRL (0, 5, 10, and 25 *μ*mol) for 24 hours. After extracting total RNA with a TRIzol reagent, RNA was turned into cDNA through the use of Taq polymerase as well as superscript reverse transcriptase via reverse chain reaction of transcription polymerase. The relative expressions of Nrf2 (5′-CATCCAGTCAGAAACCAGTGG-3′ and 5′-GCAGTCATCAAAGTACAAAGCAT-3′), HO-1 (5′-CTTCTTCACCTTCCCCAACA-3′ and 5′-ATTGCCTGGATGTGCTTTTC-3′), and NQO-1 (5′-GGGATCCACGGGGACATGAATG-3′ and 5′-ATTTGAATTCGGGCGTCTGCTG-3′) were analyzed by using RT-PCR. The combination of SYBR Green system and ViiA-7 Applied Biosystem was used to perform RT-qPCR. All genes' mRNA expression was converted into *β*-actin. The calculation of fold change across various groups was done by making use of Ct value through 2^−ΔΔCt^ (Δ^Ct^ = Ct [target gene] − Ct[*β* − actin]).

### 2.11. Statistical Analysis

For this purpose, GraphPad Prism software version 6.0 was utilized; on the other hand, three groups were compared using one-way ANOVA. All findings were expressed in the form of mean ± SD, and *p* < 0.05 was deemed statistically important.

## 3. Results

### 3.1. KRL Inhibits B[a]P-Induced Cytotoxicity

**Figure 1:**
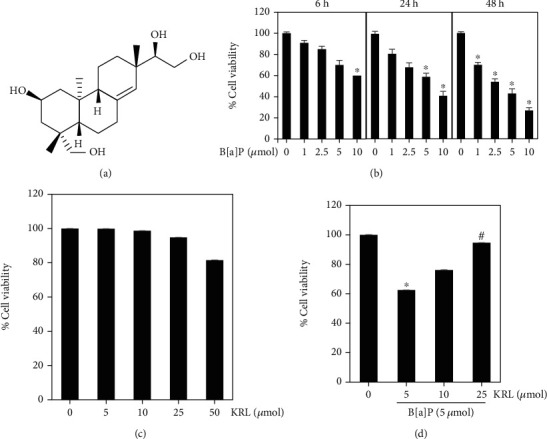
Impact of B[a]P and KRL on cell viability. (a) Chemical structure of KRL. (b) HUVECs cells were added to the different dosages of B[a]P for 6, 24, and 48 hours in various concentrations after which cell viability was determined using MTT assay. (c) Cytotoxic effect of KRL on HUVECs cells. The exposure of cells was made to varying KRL concentrations for 24 hours. (d) KRL safeguards the cytotoxic effect of B[a]P, as analyzed by MTT assay. Data is shown as the mean ± SD of triplicate values (*n* = 3), whereas ^∗^*p* < 0.05 signifies a major difference as compared to the control group. On the other hand, ^#^*p* < 0.05 denotes significant variations when compared to B[a]P alone as well as KRL with the B[a]P treatment groups.

The HUVECs' cell viability was evaluated by using the B[a]P effects for ascertaining KRL's cytotoxic potentiality. The incubation of cells was done for 6 hours, 24 hours, and 48 hours using B[a]P (Figure 1(b)). B[a]P cytotoxicity testing showed significant toxicity to HUVEC cells at different dose and times. KRL cytotoxicity testing showed that concentrations as high as 25 *μ*mol KRL were not significantly toxic to HUVECs cells (Figure 1(c)). Having said that, as illustrated in Figure 1(d), significant protection (dose-dependent) against cell death induced by B[a]P was seen with cells' preincubation using B[a]P (5 *μ*mol) and KRL (5, 10, and 25 *μ*mol).

### 3.2. B[a]P Induced Vascular Endothelial Cells ROS Inhibition by KRL

**Figure 2:**
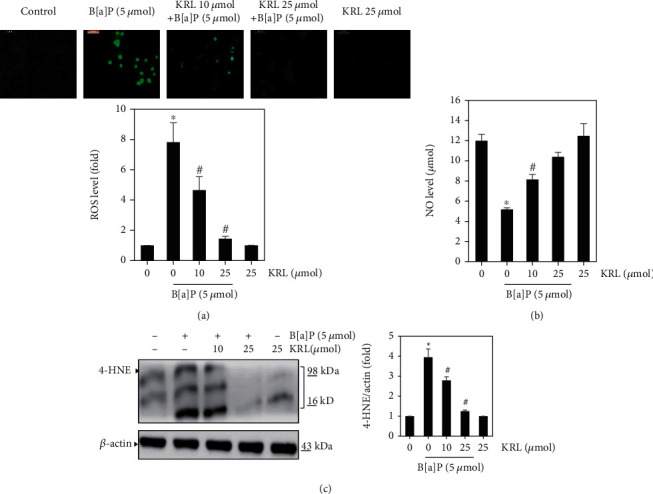
KRL inhibits ROS formation. HUVECs cells confluent at 80% were incubated with B[a]P (5 *μ*mol) and/or KRL 10 and 25 *μ*mol for 60 minutes. (a) DCFH2-DA was made use for the purpose of measuring generation of intracellular ROS. After reacting with ROS, DCFH2-DA metabolized into DCF, which, in turn, was proportionate to ROS's generation. (b) KRL effect on NO production in B[a]P-induced oxidative stress. (c) KRL on oxidative marker 4-HNE protein activation. Data was represented as the mean ± SD of triplicate values (*n* = 3), and ^∗^*p* < 0.05 represents a significant discrepancy when compared to the control group. On the other hand, ^#^*p* < 0.05 signifies major differences compared to the B[a]P alone and KRL with the B[a]P treatment groups.

Next, we used the method of DCFH2-DA fluorescent staining for ascertaining whether or not KRL could inhibit B[a]P-induced ROS generation in HUVECs cells. Figure 1(b) shows that in the context of induced cell death, ROS are the primary source of cellular death. The excessive generation of ROS has a close relationship with vascular dysfunction and cell death [4, 27]. Subsequently, we used the method of DCFH2-DA fluorescent staining for determining the curtailment ability of ROS generation induced by B[a]P.  Figure 2(a) shows a radical growth in intracellular ROS generation after B[a]P-related stimulation for one hour. The amount of free radicals generation was directly correlated to enhanced intensity of dichlorodihydrofluorescein (DCF). Nevertheless, the excessive ROS was significantly suppressed owing to KRL treatment, as Figure 2 shows. Inhibition and/or scavenging of free radicals was observed, which shows that KRL exhibits an oxidative stress induced by B[a]P as far as HUVECs endothelial cells are concerned. The improvement of endothelial dysfunction in HUVECs treated with KRL was associated with increased NO levels and was also upregulated (Figure 2(b)).

### 3.3. KRL Regulated 4-HNE Activation

ROS and by products and oxidative stress can induce various types of protein modifications such as aldehydic adducts (4-HNE and MDA). Consequently, in our study, we performed an analysis using western blot to determine whether KRL could inhibit the oxidative markers including 4-HNE. It was found that the B[a]P treatment of H9c2 cells markedly increased the accumulation of these oxidative markers. Subsequently, KRL treatment prevented this accumulation completely (Figure 2(c)). This finding demonstrates the probable effects of KRL in suppressing the elevated levels of 4-HNE.

### 3.4. B[a]P Downregulates Phosphorylation of PI3/AKT and Nrf2 Expression

**Figure 3:**
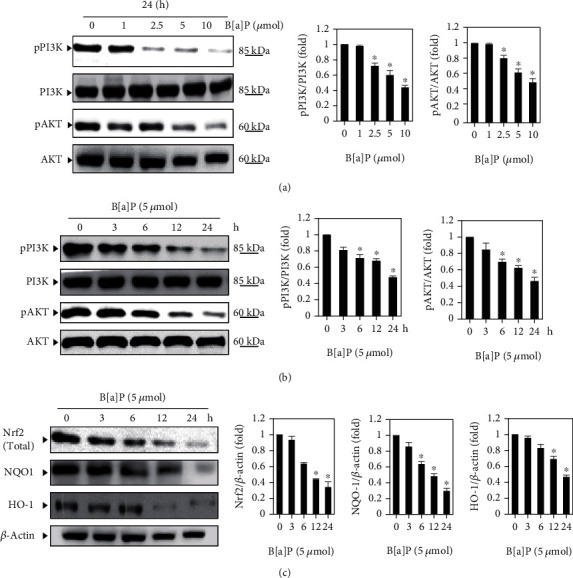
B[a]P inhibits the PI3/AKT-mediated Nrf2 signaling. (a) Cells were incubated with different concentrations of B[a]P for 24 hours, with equal amounts of whole cell lysate being exposed to SDS-PAGE. Membranes probed with anti-pPI3K and anti-pAKT antibodies and PI3K and AKT levels were deemed internal control. (b) HUVECs cells were treated with B[a]P (5 *μ*mol) for 0, 3, 6, 12, and 24 hours, and the membranes were probed with anti-pPI3K and anti-pAKT antibodies. In addition, PI3K and AKT levels were also regarded as internal control. (c) The treatment of cells with B[a]P (5 *μ*mol) for different indicated periods. After preparing extracts of total protein, western blot assessments were undertaken and analyzed Nrf2, Ho-1, and NQO-1 activations. Data is shown as the mean ± SD of triplicate values (*n* = 3), whereas ^∗^*p* < 0.05 denotes a major discrepancy when compared to the control group; on the other hand, ^#^*p* < 0.05 represents noteworthy difference when compared to the B[a]P alone and KRL with the B[a]P treatment groups.

According to several studies, ROS help activate apoptosis via pathways of PI3/AKT [28–30]. Oxidative stress is known to downregulate PI3/AKT phosphorylation and leads the inactivation of various transcription factors such as Nrf2. In order to clarify whether or not B[a]P has an impact on PI3/AKT phosphorylation expression, HUVECs were subjected to dose and time periods induced by B[a]P (Figures 3(a) and 3(b)). Through western blotting, HUVECs' exposure to B[a]P for indicated time/dose periods revealed a significant decline in the phosphorylation of PI3/AKT expression. Similarly, the expression of Nfr2 antibodies (HO-1 and NQO-1) was significantly reduced by HUVECs cells treated by B[a]P (Figure 3(c)). These results clearly show that B[a]P could play a role in inhibiting the PI3/AKT signaling which selectively targets HUVECs cells.

### 3.5. KRL Upregulates PI3/AKT Phosphorylation

Next, we sought to know if KRL induces PI3/AKT in HUVECs cells treated with B[a]P. For this reason, western blot was undertaken. The HUVECs were put into KRL (0, 5, 10, and 25 *μ*mol) and/or B[a]P incubation for 24 hours as per the findings of western blotting.

**Figure 4:**
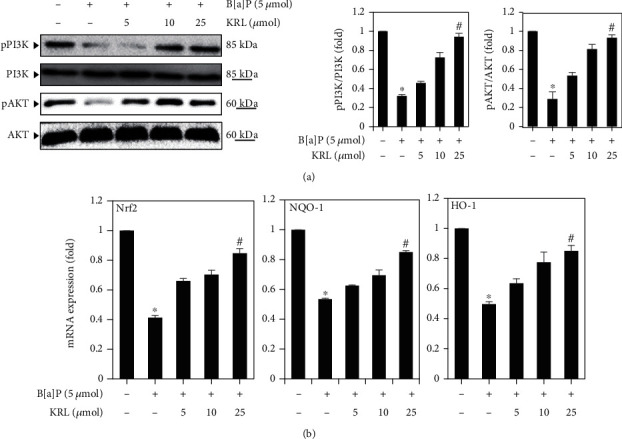
KRL activates the PI3/AKT signaling. (a) HUVECs cells were treated with B[a]P (5 *μ*mol) and/or KRL (5, 10, and 25 *μ*mol) for 24 hours. The findings of western blotting reveal the impacts of KRL on anti-pPI3K and anti-pAKT. The expression's relative ratios shown in the results of western blotting are presented below each of the results in the form of values that are relative to PI3K and AKT expression. (b) Nrf2, NQO-1, and HO-1 expression was assessed by RT-PCR. Data is represented as the mean ± SD of triplicate values (*n* = 3), and ^∗^*p* < 0.05 represents major variations as compared to the control. ^#^*p* < 0.05 meanwhile signifies noteworthy discrepancies in comparison to the B[a]P alone and KRL with the B[a]P treatment groups.

HUVECs exposure to KRL (0-25 *μ*mol) in evaluating pPI3 and pAKT expression showed that KRL enhanced the p-PI3K as well as p-AKT expression in a dose-dependent manner. However, the expression of PI3K and AKT remained almost unaltered. Therefore, the level of pPI3K and pAKT cells was significantly increased after KRL treatment. Along the same lines, KRL (0-25 *μ*mol) pretreatment and/or B[a]P (5 *μ*mol) via RT-PCR evaluation revealed high levels of mRNA concerning NQO-1, HO-1 and Nrf2, HO-1 (Figure 4(b)). At the same time, their expression was significantly higher in 25 *μ*mol KRL in comparison to others.

### 3.6. KRL Upregulates Nrf2 Signaling Proteins

**Figure 5:**
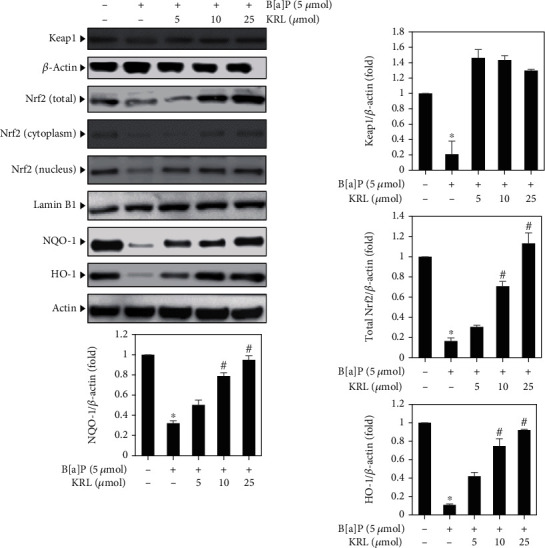
KRL upregulates the Nrf2 signaling pathways. HUVECs cell was treated with B[a]P (5 *μ*mol) and/or KRL (5, 10, and 25 *μ*mol) for 24 hours; western blotting method was used to examine what kind of impact of dose concentration had on the expressions of total, cytoplasm, and nuclear Nrf2, NQO-1, and HO-1 and antioxidant genes. In the form of internal control, *β*-actin and lamin B1 were used. Data is represented as the mean ± SD of triplicate values (*n* = 3), and ^∗^*p* < 0.05 denotes noteworthy variations in comparison to the control. ^#^*p* < 0.05 represents remarkable discrepancies when compared with the B[a]P alone and KRL with the B[a]P treatment groups.

In order to carry out further investigation of the protective mechanisms implicated via the impacts of KRL on cells treated with B[a]P, western blotting was carried out to examine protein expression levels of Kaep-1 and Nrf2 signaling proteins. We observed a reduced expression of Nrf2 and its target genes HO-1 in the B[a]P (5 *μ*mol) exposed to HUVECs cells, which was markedly reversed by KRL (10 and 25 *μ*mol), thereby upregulating the expression of Nrf2 and HO-1 in the cells (Figure 5).

### 3.7. KRL Upregulates Nrf2 Activation through the PI3/AKT Pathways

**Figure 6:**
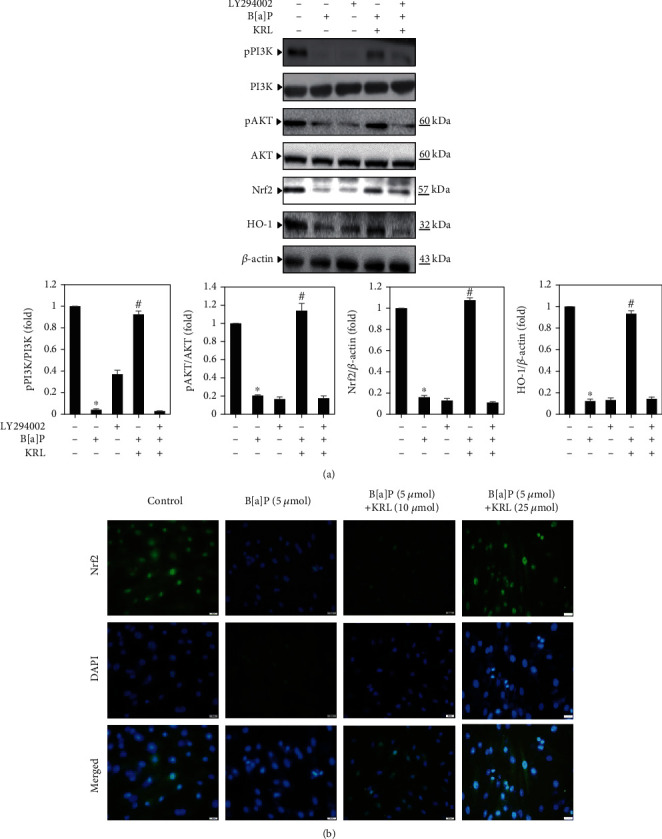
KRL can be seen to trigger the PI3/AKT pathway in B[a]P-induced HUVECs cells. (a) Cells were pretreated using a PI3K/AKT inhibitor for 2 hours. This was followed by KRL (25 *μ*mol) and/or B[a]P (5 *μ*mol) for 24 hours. Western blotting detected the pPI3K, pAKT, Nrf2, and HO-1 levels by anti-PI3K, anti-pAKT, anti-Nrf2, and anti-HO-1. (b) Nrf2 translocations. Data was represented as the mean ± SD of triplicate values (*n* = 3), whereas ^∗^*p* < 0.05 represents noteworthy discrepancies in comparison to the control. ^#^*p* < 0.05 represents major variations when compared to the B[a]P alone and KRL with the B[a]P treatment groups.

Based on above results, we undertook an examination of the manner in which KRL had an impact on oxidative stress induced by B[a]P when a PI3K/AKT inhibitor was found to be present. For ascertaining whether the pathway's activation by KRL played an important role in ensuring HUVECs cells' survival rate by modulating the expression of HO-1, we examined the kind of impact of LY294002 had on the protein expression of the aforementioned protein signals.  Figure 6(a) shows that B[a]P and/or KRL enhanced the levels of protein expression in comparison to the group that was treated with B[a]P alone, although LY294002 had largely negated this finding. LY294002 also abolished the rise in the KRL-induced levels of protein expression of all three protein signals. In addition, immunofluorescence staining showed that both B[a]P and KRL stimulated Nrf2 nuclear translocation in HUVECs cells (Figure 6(b)). However, we observed that pretreatment with KRL induced more Nrf2 nuclear translocation compared with the B[a]P treatment group.

### 3.8. Nrf2 Knockdown Attenuated the Protective Effect of KRL on HUVECs Cells under Oxidative Stress

**Figure 7:**
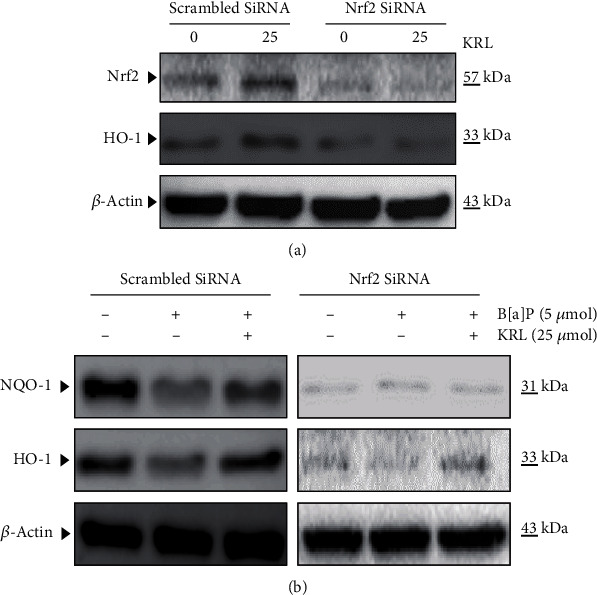
Role of Nrf2 in KRL-induced HO-1 and NQO-1 expression in endothelial cells. (a) HUVECs cells were transfected transiently with siRNA-Nrf2 and treated with 25 *μ*mol KRL. Protein was isolated and analyzed for the measurement of Nrf2 protein by western blot analysis. (b) Western blot analysis to determine the levels of NQO-1 and HO-1.

To determine the importance of regulating Nrf2, we developed a model of Nrf2 knockdown using siRNA transfection. To further elucidate the role of Nrf2 in the cytoprotective effects of KRL against oxidative stress, we transfected HUVECs cells with a Nrf2 siRNA for 24 h, then pretreated with 25 *μ*mol KRL followed by the treatment of 5 *μ*mol B[a]P. As shown in Figures 7(a) and 7(b), after the transfection with si-Nrf2 in KRL and the B[a]P treatment group, we observed markedly decreased protein expression level of Nrf2, HO-1, and NQO-1 compared with the KRL with B[a]P transfected with the scrambled group.

### 3.9. KRL Inhibits B[a]P-Induced Apoptosis HUVECs

**Figure 8:**
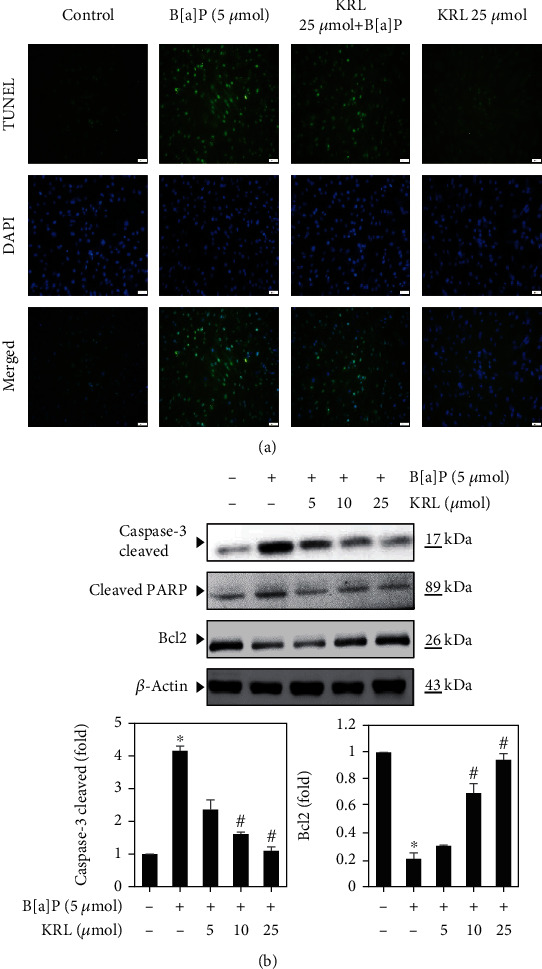
KRL suppresses DNA damage and apoptosis induced by B[a]P in HUVECs cells. (a) The pretreatment of cells was done using B[a]P (5 *μ*mol) or KRL (5, 10, and 25 *μ*mol) for a period of 24 hours and studied the TUNEL assay. (b) Cells were exposed to KRL and/or B[a]P for 24 h, and western blot was executed. Data was represented as the mean ± SD of triplicate values (*n* = 3), and ^∗^*p* < 0.05 represents noteworthy differences in comparison to control. ^#^*p* < 0.05 represents major variations in comparison to B[a]P alone and KRL with the B[a]P treatment groups.

The B[a]P-induced cell apoptosis was examined using TUNEL assay.  Figure 8(a) shows that B[a]P (5 *μ*mol) induces the TUNEL-positive nuclei in HUVECs cells. However, KRL pretreatment (25 *μ*mol) significantly reduced B[a]P-induced cell apoptosis. Therefore, KRL pretreatment safeguards HUVECs cells from oxidative stress induced by B[a]P while also preventing cell apoptosis.

### 3.10. KRL Downregulates Caspase-3 and Upregulates Bcl2 Expression

Caspase-3 plays an important role in determining apoptotic events, which includes chromatin DNA fragmentation, condensation, and apoptotic bodies' generation [31]. For this reason, our hypothesis was that the activation of caspase-3 mediates apoptosis and B[a]P-induced DNA damage.  Figure 8(b) shows that exposure of B[a]P led to an increase in the activation of caspase-3 in endothelial cells significantly, while KRL majorly curtailed the activation of caspase-3. According to our findings, the B[a]P significantly enhanced Bax expression while reducing the expression of Bcl-2 expression. On the contrary, the Bcl-2 protein level was upregulated with KRL pretreatment, while it could not downregulate the Bax expression (B[a]P-induced). Thus, it can be inferred that caspase-3's activation and the Bcl-2/Bax ratio's dysregulation mediate KRL's protective impact against apoptosis induced by B[a]P within endothelial cells.

## 4. Discussion

According to numerous studies both *in vitro* and *in vivo*, oxidative stress makes changes in homeostasis, which impairs functionality of vascular endothelia [32–34]. B[a]P, which is a major environmental carcinogen, causes DNA damage by promoting oxidative stress. This study entailed the demonstration of a significant cytoprotective effect of KRL against oxidative stress induced by B[a]P within endothelial cells. It is a known fact that the mitochondria denote a prominent source of oxygen intermediates, whereas ROS' production during the consumption of oxygen causes biological structures' oxidative damage [35]. B[a]P-triggered vascular challenge has been associated with oxidative damage. For this reason, we undertook the measurement of oxidative stress, and our study data pointed out that the treatment using B[a]P alone radically increased the levels of ROS levels when it comes to endothelial cells. Nevertheless, this impact was dose dependently and majorly inhibited owing to KRL pretreatment, which was consistent with prior studies indicating that several extracts of plants suppress formation of ROS by activating Nrf2 cascades [36].

When it comes to the regulation of the Nrf2 signaling, the signaling pathway of PI3K/AKT plays a key role [37]. For this reason, we explored the possibility of the PI3K/AKT-mediated Nrf2 signaling induced by KRL. According to our findings, the phosphorylation levels of AKT protein were notably lower in B[a]P alone-treated cells, whereas these effects significantly elevated in KRL treatment. On the other hand, significant alterations were not observed in the overall levels of AKT protein, thus implying that increasing phosphorylation of AKT protein could be a contributing factor to the Nrf2 signaling induced by KRL. We used a distinct PI3K/AKT inhibitor to treat HUVECs along with KRL for identifying the role played by the aforementioned pathway. As per our findings, when KRL was present, the PI3K/AKT signaling pathway significantly blocked the signaling.

Nrf2 enhances the responses (intracellular) to oxidative challenges [38]. Several studies have reported its beneficial impacts on many vascular ailments [39, 40]. Typically, Nrf2 can be easily degraded via the pathway of ubiquitin-proteasome. However, after it is exposed to an inducer or stressor, Nrf2 gets segregated from Keap1, moves on to the nucleus, before being sequestered with antioxidant and cytoprotective enzymes' antioxidant response facets for promoting the expression of genes [41]. According to this study, the expression of Nrf2 enhanced significantly with a corresponding rise in the duration/concentration of the treatment involving KRL. Moreover, this treatment also significantly elevated HO-1's expression. It is noteworthy that HO-1 facilitates heme's decomposition to biliverdin, iron and iron, carbon monoxide. Importantly, the products created as a result of this enzymatic reaction play a key role in cytoprotective as well as antioxidant processes via the elimination of ROS for suppressing cellular death/damage [42, 43]. Findings of this study indicated that induction of HO-1 and Nrf2 mediated by KRL could lead to the curtailment of ROS by lowering the levels of oxidative stress within HUVECs cells. At the same time, this study entailed the use of NAC, which is a popular ROS scavenger, for treating HUVECs in the form of positive control. In turn, this significantly lowered overproduction of ROS induced by B[a]P; the extent of which was similar to that of treatment using KRL.

Apoptosis is predicated on the balance between anti and proapoptotic proteins. In this context, Bcl-2 family proteins regulate apoptosis in the capacity of inhibitors (Bcl-2) or inducers (Bax). The exposure of HUVECs to B[a]P in this study for 24 hours was denoted by a higher rise of Bax/Bcl-2 ratio, thus indicating apoptosis induction. Enhanced apoptosis disrupts function of endothelial cell and atherosclerosis's progression, and possibly increases the instability associated with atherosclerotic plaques. Moreover, this study also revealed that KRL decreased the aforementioned ratio to possibly attenuate the endothelial cell's apoptotic death induced by B[a]P. Consistent with our results, another study had revealed that male sperm cells' death was caused by B[a]P exposure, more particularly by getting the Bax/Bcl-2 signaling activated. Another observation of that study was that this phenomenon is linked to abnormal stress (mediated by ROS) and inflammation [44]. In addition to getting the Bax/Bcl-2 ratio's dysregulation attenuated, the extracts of KRL are also likely to prevent myocardial cells' apoptotic death among animals that have reached an advanced age [45]. KRL's cytoprotective impact is linked to enhanced activity of antioxidants and lower oxidative stress. Therefore, inference of these findings is that KRL possibly prevents apoptosis (B[a]P-induced) via enhanced status of antioxidants.

## 5. Conclusion

In conclusions, this is the first study demonstrating that pretreatment with KRL could alleviate B[a]P-induced oxidative stress in human umbilical vein endothelial cells by improving the antioxidant capacity and activating the Nrf2 signaling via the PI3/AKT pathway.

## Data Availability

The data that support the findings of this study are available from the corresponding author upon reasonable request.
